# Accurate Predictive Modeling of Conservation Status in Animal Species Using Supervised Learning

**DOI:** 10.1002/ece3.72157

**Published:** 2025-09-15

**Authors:** Anais Aoki, Arun Sethuraman

**Affiliations:** ^1^ Department of Biology San Diego State University San Diego California USA; ^2^ Western Ecological Research Center ‐ San Diego Field Station US Geological Survey San Diego California USA

## Abstract

Conservation management to mitigate extinction of wildlife becomes more crucial than ever as global impacts due to anthropogenic activities and climate change continue to create devastation for species around the globe. The International Union of Conservation of Nature (IUCN) RedList does not currently utilize genetic information to assess species conservation status despite the availability of molecular data. Here we use over 7300 animal studies collated from the MacroPopGen database, and over 450 published articles from the public repository DataDryad, focused on conservation and population genetics, sampling across a variety of invertebrate and vertebrate taxa, and using IUCN classifications to predict species endangerment across and within animal taxa using machine learning. We test hypotheses and show significant (*p* < 0.05) (a) decreased genetic diversity, and (b) increased genetic differentiation in bird and fish taxa with increased degree of endangerment. Additionally, our models were able to accurately (overall accuracy of 93.16%) predict species threat levels classified by the IUCN using both measures of genetic diversity and differentiation with IUCN assessment criteria. We propose that future studies that assess conservation status of animal taxa utilize a combination of predictor variables, that include available genomic data, along with demographic, phenotypic, and census data.

## Graphical abstract

In this manuscript, we describe (1) a literature survey of current conservation genetics literature to test hypotheses of decreased genetic diversity and increased genetic differentiation (due to fragmentation and isolation) with increased threat status, and (2) build a predictive random forest model for supervised learning to accurately classify threat status of animal species by training on a variety of genetic, behavioral, geographical, biotic/abiotic stressors, and other variables reported by the IUCN. Our models show high levels of accuracy in predicting threat status across all species classes analyzed (> 90%), and can therefore be used as an effective way to assess conservation status.

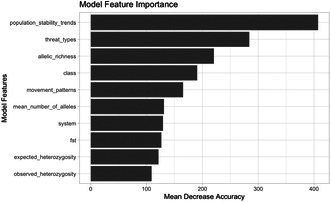

## 1 Introduction

The conservation of species across the globe grows more urgent as anthropogenic activities and climate change continue to add pressure on species and their natural environments (summarized in Schiebelhut et al. 2024). These anthropogenic stressors come at a rate at which species simply cannot adapt fast enough, changing their natural selective pressures and evolutionary potential (Garner et al. 2005). Current extinction rates of species are above background extinction rates, with mammalian and vertebrate species having extinction rates upwards of 100 times as fast as other taxonomic groups (Ceballos et al. 2015). Anthropogenic activity not only affects individual groups of species but is harmful to the ecosystem as a collective network, potentially leading to coextinction events (Morris 2010).

Genetic diversity is a critical component in the survival of different species and is a determinant of a population's ability to adapt and persist in a changing environment (Garner et al. 2005). Endangered populations typically undergo a process of population decline and fragmentation, where genetic drift is expected to have a stronger effect than selection and gene flow. As a result, these small and isolated populations become susceptible to reductions in genetic diversity, where slightly deleterious alleles might increase in frequency or become fixed within a population, increasing homozygosity as a consequence, and in turn reducing heterozygote advantage (Hansson and Westerberg 2002). This also increases opportunities for inbreeding and in turn can also further compromise the viability of these populations.

Conservation geneticists have long utilized population genetic information and estimation of genomic summary statistics to measure genetic diversity to assess species of conservation concern (DeWoody et al. 2021). There are different types of genetic diversity measures utilized by population geneticists to study gene variations and their frequencies in natural populations. Heterozygosity is one of the most used measures of genetic diversity and is usually one of the first reported measures in a study. Effective population size (Ne) is another measure used to quantify how genetic diversity declines within a population. Smaller Ne is often associated with higher population risk (Lonsinger et al. 2018). Allelic richness, which provides a measure to predict allele recovery in a population and is crucial for a species evolutionary potential, has been shown to be a more sensitive measure than heterozygosity in such scenarios (Greenbaum et al. 2014). Endangered populations become more structured due to isolation, and therefore Wright's fixation index, *F*st, is a widely used measure of genetic differentiation to quantify how diverged two populations have become due to the lack of gene flow between them. A recent study looked at over 27,000 terrestrial vertebrate species and found significant population decline and range shrinkage when comparing historic and present‐day geographic ranges, with terrestrial species having shown a loss of over 40%, even those classified as “low concern” (Ceballos et al. 2015).

Now more than ever, with the continued advancements in third generation sequencing technologies (TGS), genomic capabilities and our understanding of the genome proceed to progress. TGS makes detection of species evolution on recent time scales possible, providing insights into the development of new zoonotic diseases (Xiao and Zhou 2020) and responses to global climate change and anthropogenic pressures. With ever‐expanding technology availability, population genetics simulation studies have also increased, helping researchers investigate the effects of different genetic models on large‐scale data (Yuan et al. 2012). Furthermore, data repositories and genome database sites such as DataDryad, ENSEMBL, and NCBI serve as great resources in data availability for public research.

However, despite ongoing efforts, recent studies show that there lies a strong disconnect between the knowledge obtained from genetic research and its incorporation in conservation management plans and policies (Tobias et al. 2025; Britt et al. 2018; Sandström et al. 2019; Taylor et al. 2017; Ottewell et al. 2015). This is known as the “Conservation Gap.” This can be attributed to issues such as practitioners' accessibility to data, lack of communication and clear outlines of proposed management plans, hesitation to allocate funding for research, as well as a lack of research in non‐model systems (Britt et al. 2018; Sandström et al. 2019; Taylor et al. 2017; Ottewell et al. 2015). In a meta‐analysis looking at conservation studies, researchers found that 66% of studies were on species that were of low conservation concern and observed that only 38% of studies specifically identified how their analyses could inform policy decisions through clearly stated recommendations of conservation management plans (Britt et al. 2018).

The International Union for Conservation of Nature, the IUCN, is a globally recognized organization that works with collaborators in its efforts to monitor and sustain biodiversity across the globe by using a set of criteria to categorize and maintain a ranking of species, known as their Red List. The Red List is widely used as an indicator to determine extinction risk for a wide range of species across all taxonomic groups and is commonly reported in scientific studies. The IUCN ranks species by estimating census size based on 60 to 70 criteria such as population decline, range extent and occupancy, and estimated number of mature adults (IUCN 2021). They do not currently utilize measures of genetic diversity and differentiation when assessing species on their Red List. In a recent study, researchers tested these criteria on previously published articles to determine which criteria were most effective in determining conservation status (Willoughby et al. 2015). They found that these criteria did not effectively identify populations with low genetic diversity proposed a novel approach which were integrates IUCN's evaluation of census size, along with incorporating effective population size (Ne) based on biological data available, to estimate the number of generations until heterozygosity is analyzed by 25% as a way to determine conservation status. They do not suggest genetic diversity as the primary criterion for species ranking; however, they suggest finding ways to incorporate genetic diversity that they measure with current IUCN Red List ranking criteria due to the availability of molecular data (Willoughby et al. 2015). Another recent study, looking at three types of genetic data, mitochondrial, microsatellites, and whole genomes, found that the genetic diversity system did not accurately identify species classified as threatened on the IUCN's RedList, indicating that this metric is not a good indicator for using genetic diversity to inform conservation policy. They conclude there is a need to develop a metric that is designed to assess and incorporate genetic diversity (Schmidt et al. 2023).

Here, we propose an alternate approach to predicting the conservation status of animal taxa that incorporates both measures of genetic diversity and differentiation, as well as ecological characteristics of a species and its population distributions evaluated by the IUCN in a machine learning framework. We hypothesize that across taxa, increased endangerment (measured via IUCN RedList indicators) will be strongly associated with (a) a decrease in overall genetic diversity and (b) increased genetic differentiation. We also hypothesize that genetic variables such as genetic diversity and differentiation will be accurate predictors of RedList status. We specifically chose animal taxa in this study, owing to the wealth of genetic data available across animals in the conservation literature, as compared to plants, but the methods described herein are easily extensible to other taxa.

## 2 Methods

### 2.1 Literature Sampling and Data Collection

Measures of genetic diversity (observed and expected heterozygosity, allelic richness and mean number of alleles) and genetic differentiation (*F*st) were collated from over 450 published articles that focused specifically on conservation and population genetics, from the repository DataDryad (www.datadryad.org). These data include both vertebrate and invertebrate taxa, consisting of marine and land mammals, birds, fish, amphibians, and reptiles. The species in these studies spread across various geographic ranges and habitats, including species from island populations across all continents and occupying regions of both the Northern and Southern hemispheres. All studies included in the analysis were published between the years 2010 and 2022 and utilized microsatellites (Table 1). Average heterozygosity and mean number of alleles were calculated from raw data for studies that did not explicitly report them. Global *F*st estimates were recorded when reported, and the more conservative values (i.e., at least as differentiated as the reported conservative *F*st value) were recorded from studies that listed a range (pairwise analysis) to avoid overestimation of differentiation. Similarly, for genetic diversity measures, the upper limit of values was recorded to avoid underestimation of genetic diversity in order to ensure conservative inference of genetic diversity comparisons among threatened species (Figure 3). No outliers were removed from the dataset. Using the IUCN's Red List database (2021), each species IUCN Red List ranking was logged, along with species population stability trends, number of mature adults, habitat system and migration patterns, number of threats and trade uses listed for each species.

**TABLE 1:** Sample sizes for studies from the literature, surveyed from DataDryad (2010–2022).

Class	Number of species	Heterozygosity	*F*st	Allelic richness	Mean alleles
Mammalia	118	99	54	55	54
Aves	110	76	40	52	37
Actinoptergii	75	60	29	49	42
Amphibia	26	10	4	8	3
Reptilia	16	14	6	5	11

*Note:* Not all studies reported each measure of genetic diversity or differentiation.

### 2.2 Data Appraisal and Statistical Analysis

The data were first evaluated for normality and homogeneity of variance across each taxonomic class, for each measure of genetic diversity and differentiation using R v. 4.1.1 (RCore Team 2021). The Shapiro‐Wilks' test was performed to test for normality, and Levene's test was performed to check for homogeneity of variance (Tables 2, 3). To assess mean differences in genetic diversity measures and differentiation across each taxonomic grouping according to their IUCN Red List ranking, a one‐way ANOVA test was performed at a false positive rate of 0.05 (Table 4). For the ANOVA tests that were statistically significant, Tukey's HSD post hoc test was run to evaluate the significance between each Red List ranking. Using a critical value of 0.05, the Kruskal‐Wallis nonparametric test was run on data that failed normality after log transformation. Due to the imbalance of studies that were categorized as vulnerable, endangered, and critically endangered, one‐way ANOVA analysis was only evaluated on mammals, fish, and birds for microsatellite data. Species threat level, classified by the IUCN was also evaluated to further assess mean differences in genetic diversity measures and differentiation based on their reported conservation status (Table 5). The IUCN classifies species ranked as least concern and near threatened as non‐threatened species, and classifies species ranked as vulnerable, endangered, and critically endangered as threatened species. Welch's two‐sample t‐tests, with a critical value of 0.05, were performed on all taxonomic groups to evaluate mean differences in *F*st, allelic richness, and mean number of alleles among species threat level. To accurately assess the relationships between species genetic diversity and their IUCN status, the phylogenetic history between species from the same class was evaluated to determine how much of their standing genetic diversity and differentiation is influenced by shared ancestry. All species taxonomy was cataloged using the Catalog of Life Database (www.catalogueoflife.org). Phylogenetic trees were then constructed for each of the five classes, and phylogenetic inferences were made using the *phytools* package in R to quantify correlations across species classes and IUCN Red List ranking for each measure of genetic diversity and differentiation. We then tested two hypotheses to understand the contribution of genetic diversity and differentiation measures to species threat status – (1) H0: Genetic diversity variables decrease with increased threat levels, and (2) H0: Genetic differentiation variables increase with increased threat levels (with increased geographic isolation and local inbreeding).

**TABLE 2:** Shapiro‐Wilks tests of normality in genetic diversity (observed heterozygosity, allelic richness, mean number of alleles) and genetic differentiation (*F*st) measured across all animal taxa from over 450 studies.

Class	Ho (observed heterozygosity)	Ar (allelic richness)	Na (mean number of alleles)	*F*st (genetic differentiation)
Mammalia	*p* = 1.798e‐08 Failed w/log transformation	*p* = 0.4848 Passed w/log transformation	*p* = 0.09953	*p* = 0.1918 Passed w/log transformation
Aves	*p* = 0.5288	*p* = 0.0691 Passed w/log transformation	*p* = 0.0293 Failed w/log transformation	*p* = 2.04e‐16 cannot log transform, failed normally
Actinopterygii	*p* = 1.5e‐06 failed w/log transformation	*p* = 0.9055 Passed w/log transformation	*p* = 0.4303 passed w/log transformation	*p* = 3.53 e‐08 cannot log transform, failed normally
Reptilia	*p* = 0.0593	*p* = 0.2973	*p* = 0.8949	*p* = 0.9942
Amphibia	*p* = 0.9009	*p* = 0.4941	*p* = 0.4519	*p* = 0.977

**TABLE 3:** Levene's Homogeneity of Variance Test in genetic diversity (observed heterozygosity, allelic richness, mean number of alleles) and genetic differentiation (*F*st) measured across all animal taxa from over 450 studies.

Class	Ho (observed heterozygosity)	Ar (allelic richness)	Na (mean number of alleles)	*F*st (genetic differentiation)
Mammalia	*p* = 0.4785	*p* = 0.4159	*p* = 0.5314	*p* = 0.4643 Passed with log transformation
Aves	*p* = 0.0548	*p* =0.1745	*p* = 0.486	*p* = 0.2032
Actinopterygii	*p* = 0.3021	*p* = 0.4667	*p* = 0.8515	*p* = 0.688
Reptilia	*p* = 0.5161	*p* = 0.5866	*p* = 0.5488	*p* = 0.5673
Amphibia	*p* = 0.339	n/a	*p* = 0.5787	*p* = 0.1386

**TABLE 4:** One‐Way ANOVA/Kruskal—Wallis tests of significant differences in genetic diversity measures (observed heterozygosity, allelic richness, mean number of alleles) and genetic differentiation (*F*st) across all animal taxa, reported from over 450 collated studies. The H0 tested was no differences among IUCN Red List rankings within each class.

Class	Ho (observed heterozygosity)	Ar (allelic richness)	Na (mean number of alleles)	*F*st (genetic differentiation)
Mammalia	*p* = 0.3267*	*p* = 0.612	*p* = 0.840	*p* = 0.139
Aves	*p* = 0.161	*p* = 0.303	*p* = 0.0003*	*p* = 0.0689*
Actinopterygii	*p* = 0.294	*p* = 0.098	*p* = 0.0113 Tukey *p* = 0.0266 Least concern/endangered	*p* = 0.681*

*Note:* Kruskal‐Wallis tests were run on data that did not pass normality after transformation (*p*‐values shown for non‐parametric Kruskal‐Wallis tests shown with asterisk).

**TABLE 5:** Two sample t‐test/Mann–Whitney tests of significant differences in genetic diversity measures (observed heterozygosity, allelic richness, mean number of alleles) and genetic differentiation (*F*st) across all animal taxa, reported from over 450 collated studies. The H0 tested was no differences between threat level (Non‐threatened vs. threatened) within each class.

Class	Ho (observed heterozygosity)	Ar (allelic richness)	Na (mean number of alleles)	*F*st (genetic differentiation)
Mammalia	*p* = 0.0468*	*p* = 0.04669	*p* = 0.9477	*p* = 0.0375
Aves	*p* = 0.01286	*p* = 0.1438*	*p* = 7.14e‐06*	*p* = 0.0272*
Actinopterygii	*p* = 0.1276*	*p* = 0.2005	*p* = 0.00065	*p* = 0.2576*
Reptilia	*p* = 0.3451	*p* = 0.9176	*p* = 0.139	*p* = 0.2475
Amphibia	*p* = 0.0434	n/a	*p* = 0.3386	*p* = 0.9402

*Note:* Non‐parametric Mann–Whitney tests were run on data that failed normality, *p*‐values shown with an asterisk. Also note, Aves allelic richness was run as a nonparametric test due to non‐normality after log transformation.

### 2.3 Predictive Modeling

To infer conservation status across animal phyla, supervised machine learning modeling methods were implemented using a random forest algorithm. The model was trained and tested using the data from the literature survey, as well as the integration of an additional 7538 data entries from the MacroPopGen database with species sampled from the Americas (Lawrence et al. 2019) (Table 6). This database was chosen owing to its recentness and comprehensive nature in collating genetic data from across animal taxa.

**TABLE 6:** Combined statistics on the number of non‐threatened (IUCN RedList rankings—Least Concern, Near Threatened, Vulnerable) and threatened (IUCN RedList rankings—Endangered, critically endangered) across the 450+ studies collated from DataDryad and the MacroPopGen database, separated by class.

Class	Non‐threatened	Threatened
Actinopterygii	1921	370
Amphibia	890	164
Aves	646	159
Mammalia	1744	343
Reptilia	702	599

There are two main attribute categories included in the model: (1) genetic summary statistics, and (2) IUCN assessment criteria and reported features. The genetic summary statistics incorporated into the model included measures of genetic differentiation (*F*st), genetic diversity, consisting of observed and expected heterozygosity (Ho and He, respectively), mean number of alleles (Na), and allelic richness (Ar). IUCN assessment criteria and reported features include species population stability trends and species habitat systems, which list one or more of the following: terrestrial, marine, or freshwater; movement patterns, which list one of the following: not a migrant, attitudinal migrant, full migrant, or nomadic included (IUCN 2021). IUCN reported threats and species commercial use and trade were summarized into counts.

To partition the data, test and train the model, and generate model accuracy and confusions matrices, the *randomForest* package was used in R (R CoreTeam 2021). The results were then visualized using the *ggplot2* package. Due to inconsistent patterns among genetic diversity measures and *F*st across all classes, all the collated data were first run in one predictive model, with species threat level (non‐threatened vs. threatened) as the response variable (Table 7). Five additional models were also generated, separating the data by species class. Due to the unbalanced nature of the data, the data was first imputed to infer missing data for each attribute (Table 8). The imputed data was partitioned into two groups, a training and test set for the model, with a split ratio of 70/30 respectively. Confusion matrices were generated for both the test and training sets to determine how accurately the model classified each species according to their threat level. For each model, the optimal number of variables for each internal node of the tree was checked by comparing the position of the lowest out‐of‐bag error rate in the vector with a length of 10. The optimal number of total trees that the random forest algorithm should generate was checked by plotting the error rate of the random forest, with the number of trees on the x‐axis, and the error rate on the y‐axis. It was decided that the error rates of each classification from the training and test sets, along with the random forest out‐of‐bag error rates showed minimal change in decline after sampling 300 trees. The varImPlot() function was called to generate the importance plots for the model, which estimated the mean decrease in accuracy by iteratively excluding each feature, and the mean decrease in impurity, given by the Gini index, a coefficient that illustrates how each feature contributes to the homogeneity of the nodes and leaves in the random forest. To obtain the marginal effect that each genetic diversity measure has on the probability that the model classifies species threat level, the *partialPlot()* function was called.

**TABLE 7:** Random forest model results including data from all five classes across the 450+ studies collated from Data Dryad and the MacroPopGen database.

Top 3 model attributes	Out of bag (OOB) error	Test accuracy	Balanced accuracy	Sensitivity (non‐threatened)	Specificity (threatened)
Population stability trends, threat types, allelic richness	3.69%	96.32% (CI: 95.47%–97.05%)	93.16%	98.94%	87.38%

**TABLE 8:** Random forest model results by species class.

Class	Top 3 model attributes	Out‐of‐bag error rate	Test accuracy/95% CI	Balanced accuracy	Sensitivity (non‐threatened)	Specificity (threatened)
Actinopterygii	Number of threats, population stability, and allelic richness	2.57%	96.42% [CI: 94.76%–97.67%]	90.48%	99.65%	81.30%
Amphibia	*F*st, population stability, number of threats listed	3.33%	94.91% [CI: 91.98–97.01]	90.57%	96.82%	84.31%
Aves	Number of threats listed, movement pattern, and mean alleles	9.06%	92.15% [CI: 88.01–95.21]	82.08%	96.59%	67.57%
Mammalia	Population stability, trade use, and movement pattern	3.19%	97.21% [CI: 95.63–98.34]	92.07%	99.45%	84.69%
Reptilia	Population stability, number of threats listed, and allelic richness	2.68%	97.78% [CI: 95.82–98.98]	97.91%	96.96%	98.86%

## 3 Results

### 3.1 Review of the Conservation Genetics Literature

**FIGURE 1:**
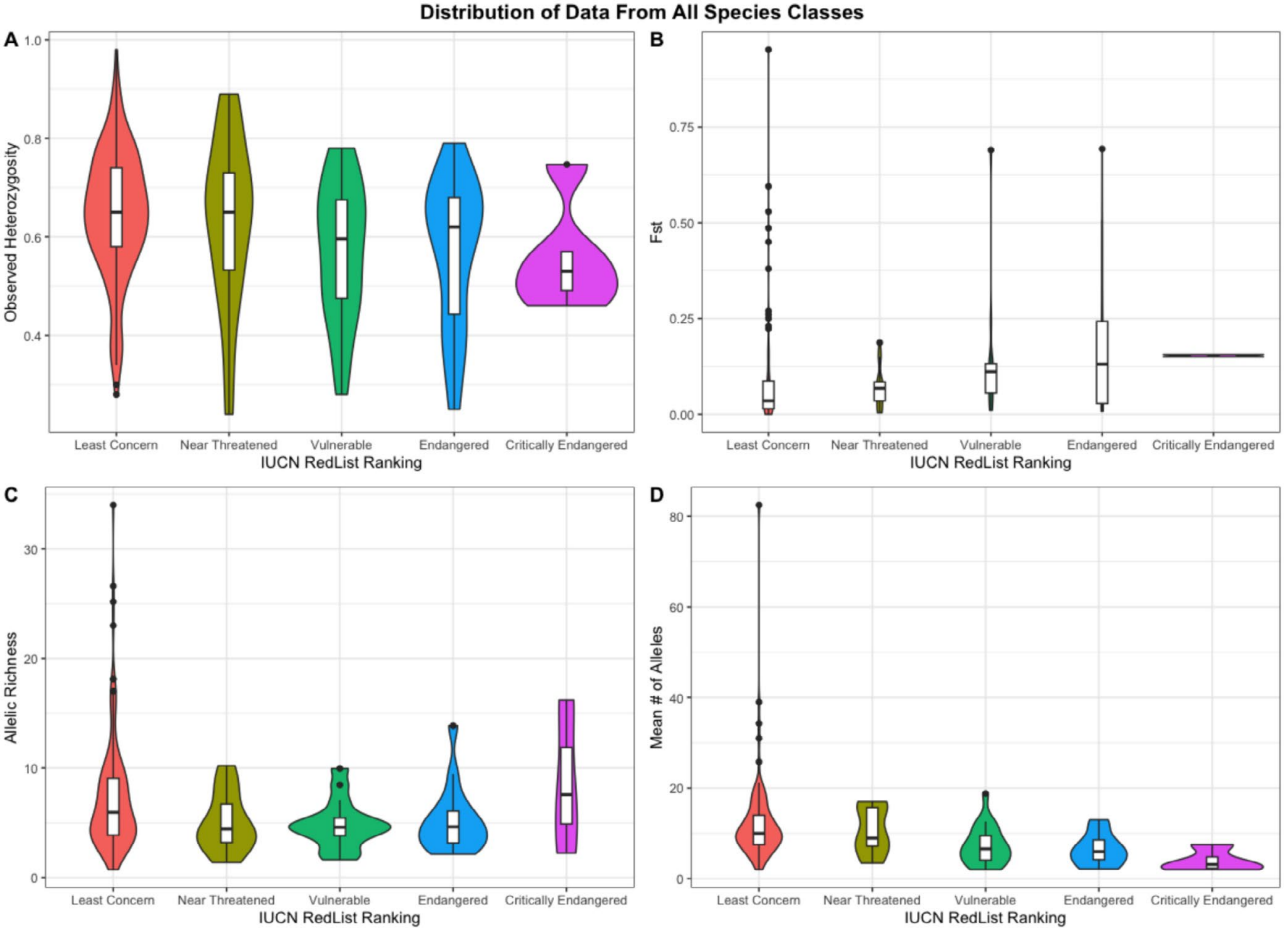
Distributions of genetic diversity (observed heterozygosity, allelic richness, and mean number of alleles) and genetic differentiation (fixation index, *F*st) across microsatellite studies collated from 2010 to 2022 in our literature survey, classified by IUCN RedList rankings (Least Concern—Critically Endangered).

Overall, measures of genetic diversity were significantly lower in species classified as threatened, compared to non‐threatened. Whereas genetic differentiation was significantly higher in species classified as threatened compared to those classified as nonthreatened (Figure 1). Looking at differences in genetic diversity measures across IUCN Red List ranking, mean number of alleles was significantly higher in species classified as least concern than in those classified as endangered (*p* = 0.0113, df = 4) in fish (Table 4). The Kruskal‐Wallis test determined that there were also significant differences in mean number of alleles (*p* = 0.0003, df = 4) in birds (Table 4). There were significant differences in observed genetic diversity measures when comparing species threat level classified by the IUCN. Observed heterozygosity was significantly lower in mammals (*p* = 0.0468), birds (*p* = 0.0128), and amphibians (*p* = 0.0434) classified as threatened compared to non‐threatened (Table 5). There were only significant differences in allelic richness when comparing species threat level for mammals (*p* = 0.0466) (Table 5). When comparing mean number of alleles with species threat level, there were significant differences in birds (*p* = 7.14e‐06) and fish (*p* = 6.5e‐04) (Table 5; Figure S1).

### 3.2 Supervised Machine Learning

**FIGURE 2:**
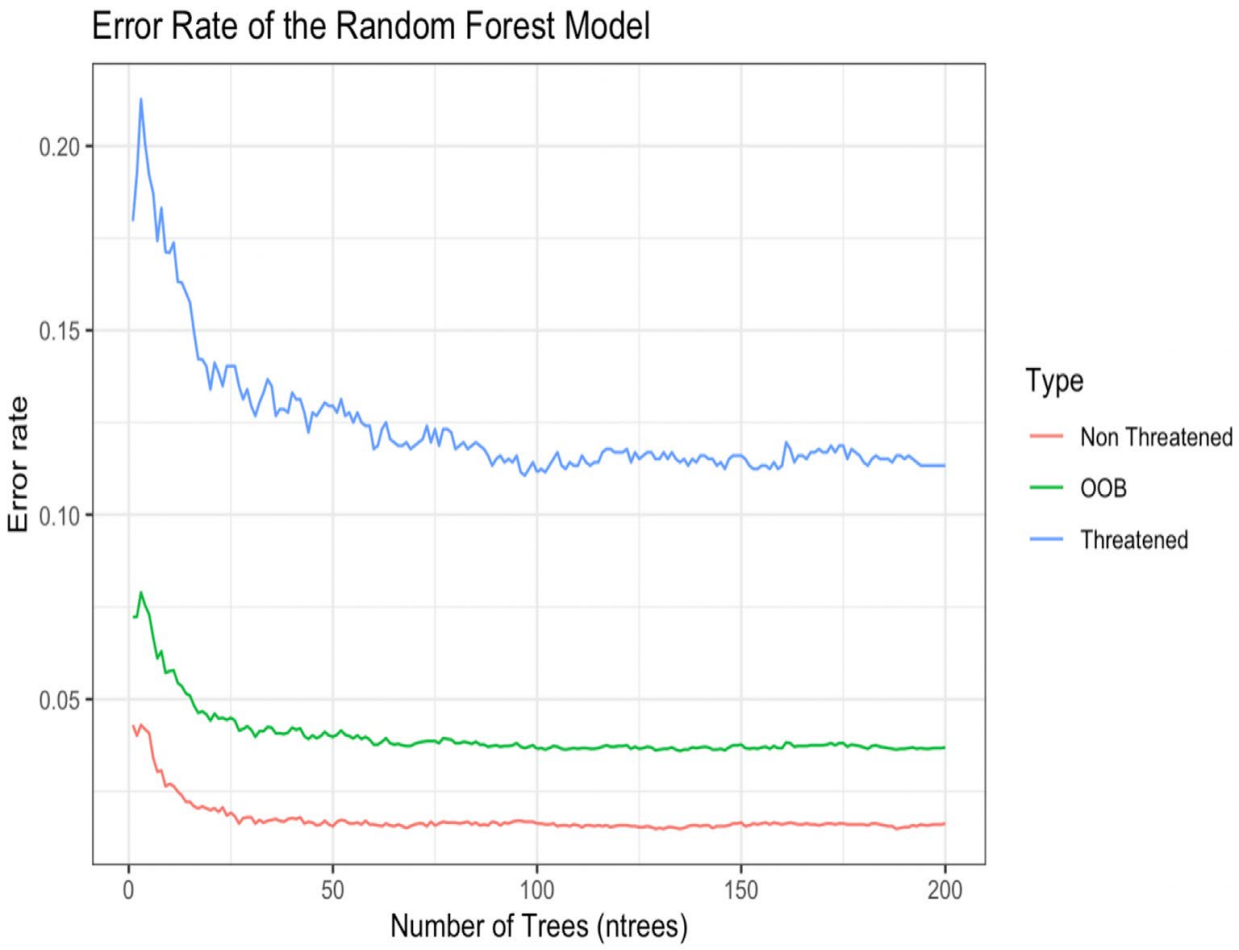
Error rate of the out‐of‐bag estimator and response variables (threat level) of the random forest model including data from all five classes.

**FIGURE 3:**
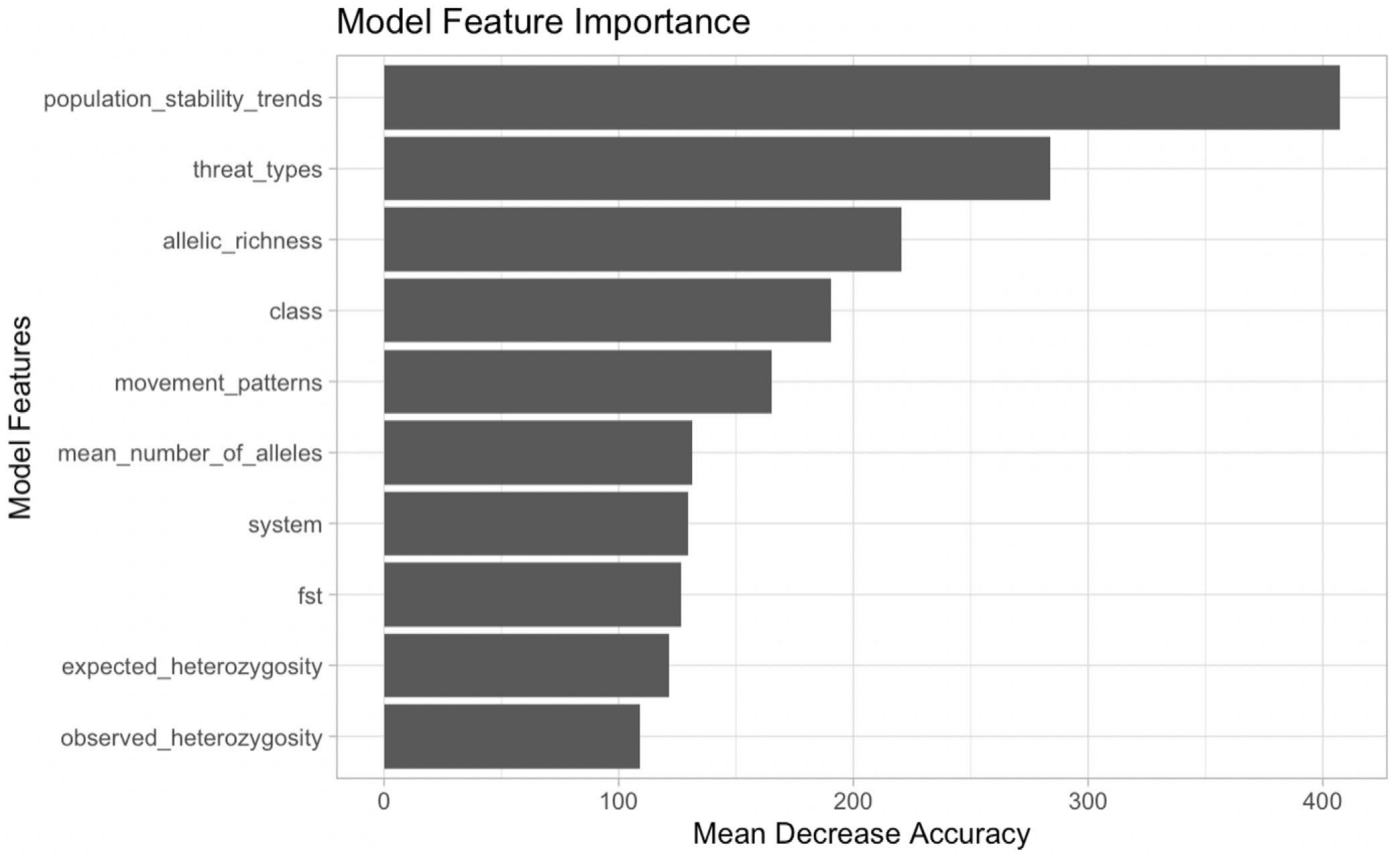
Model attributes that contribute to the greatest mean decrease in accuracy in the random forest model of threat level.

The response variable for the random forest classification model was species threat level, with non‐threatened species sorted as the positive class and threatened species sorted as the negative class. The accuracy of the model was validated using the out‐of‐bag estimator, which estimated an error rate of 3.69%, with a class error of 1.63% for non‐threatened species and 11.33% for threatened species (Figure 2). The test classification accuracy of the random forest model was 96.32% (CI: 95.47%–97.05%), with a positive prediction value of 96.40% and a negative prediction value of 96.01%. The test classification had a sensitivity score of 98.94% and a specificity score of 87.38%. This model had an overall balanced accuracy score of 93.16% (Table 7). The three most important features of the model were population stability trends (given by the IUCN), followed by the number of threats (listed by the IUCN, included as counts), and allelic richness (reported by each study) (Figure 3).

### 3.3 Predictive Models by Species Class

When generating models for each species class, population stability trends were the most consistently important attribute for all the models, followed by the number of threat types (non‐threatened vs. threatened) reported by the IUCN (Figure 2). Rankings of feature importance from genetic diversity measures and genetic differentiation all differed across each species model (Table 8, Figures S2–S11). Genetic differentiation was the most important model attribute for the class Amphibia model. Mean number of alleles was the third most important attribute for the class Aves model, and allelic richness was the third most important model attribute for the models of class Actinopterygii and Reptilia (Table 8).

The model for class Reptilia was the best fitting model, which yielded the highest test accuracy of 97.78% (CI: 0.9582–0.9898), and an overall balanced model accuracy of 97.71%. This model was the only model that was able to accurately classify both threatened and non‐threatened species, with a class error of 3.18% for non‐threatened species, and 2.12% for threatened species (Table 8). Each of the other class model's accuracies suffered most due to misclassification of threatened species compared to non‐threatened species. The class Mammalia was the next best fitting model with a test accuracy of 97.21% (CI: 95.63–98.34), and an overall balanced model accuracy of 92.07%, followed by class Actinopterygii with a test accuracy of 96.42% (CI: 4.76–97.67), and an overall balanced model accuracy of 90.48%, and class Amphibia, with a test accuracy of 94.91% (CI: 91.98–97.01), and an overall balanced model accuracy of 90.57% (Table 8). The model for class Aves had the lowest overall model accuracy out of the five models, with a test accuracy of 92.15% (CI: 88.01–95.21), and a balanced accuracy of 82.08%. The model for class Aves was the only model that did not have population stability trend as a top attribute when it came to feature importance, but rather was influenced by movement pattern (Figure S8).

## 4 Discussion

One of the most predominantly used extinction risk indicators is the IUCN's Red List, which does not currently utilize genetic estimates of population genetic diversity and differentiation when assessing species conservation status. Monitoring and sustaining biodiversity continue to be a global concern with increasing pressures on species due to anthropogenic activity. On July 12th, 2021, the UN Convention on Biological Diversity (CBD) released a global biodiversity framework along with four goals to be achieved by 2050. Goal A aims to reduce the extinction rate and risk of species by 10‐fold and to maintain the genetic diversity of both wild and domesticated species. In order to track progress on this goal, along with working to close the gap between research and policy, the threat status of species must be accurately classified and communicated. Here, we develop a machine learning framework to accurately predict threat status based on a combination of genetic diversity, differentiation, and a host of behavioral, abiotic threat, ecological, and geographical variables.

### 4.1 Random Forest Model Accuracy

The test accuracy of the first model, including species from all five animal classes, significantly improved with the integration of the larger MacroPopGen dataset. However, due to the unbalanced nature of both datasets overall, missing measures of genetic diversity and differentiation needed to be imputed to retain the entire dataset for prediction. Unfortunately, when dealing with large‐scale biodiversity datasets, missing or incomplete data is a common problem, especially when dealing with spatial and temporal biases (Boakes et al. 2010; Bowler et al. 2024). Although random forest models have high‐quality imputation methods, finding better proxies for missing data, such as through simulation studies, could help limit misclassifying species with low genetic diversity (Epperson et al. 2010; Peng et al. 2014). Across all models, except for the model for class Reptilia, the overall test accuracy suffered most from the misclassification of threatened species compared to non‐threatened species (Table 8). Class Reptilia was the only subset of data that had a more balanced set of species that were classified as threatened to those classified as non‐threatened. The other four classes included three to four times more species that were classified as non‐threatened compared to threatened (Table 6). Overall, the models performed most optimally when separated by species class (Table 8). This is likely due to differences in average genetic diversity measures as well as ecological differences by virtue of being more dissimilar to other members of different classes compared to their own.

### 4.2 Integration of Genetic Diversity Measures and Differentiation With IUCN Criteria

The goal of the machine learning framework was to evaluate the possibility of incorporating summary statistics from genetic data with IUCN assessment criteria in efforts to bridge the conservation gap between research generated by practitioners and its incorporation into policy decisions. Population stability trends and the number of threats listed, both given by the IUCN, were determined to be the most important feature attributes across all models. Although ranked lower in model attribute importance, genetic differentiation (*F*st) and measures of genetic diversity, such as the mean number of alleles and allelic richness, indicated having attribute importance in the random forest models as well.

For future investigations, more class‐specific attributes, or features such as geographic ranges and occupancy can be explored to incorporate into these models. With the notion that these models can always be improved upon, we believe that this is a realistic and simplistic approach that can streamline how genetic data are communicated and interpreted. Practitioners can utilize these models to make inferences on the conservation status with newly generated data by using the *predict* function to assess threat level and generate a confusion matrix by calling the *confusion matrix* function to generate prediction accuracy scores. Furthermore, even though differentiating between low and decreasing genetic diversity can be challenging, the partial dependence plots generated by each model should be referenced in tandem with prediction outputs to help understand the baseline for what is considered to be low or high genetic diversity with the data available. At the very least, these predictive models can serve as a proxy for species whose IUCN status has not been as recently updated to still make inferences on conservation status based on information that is presently available. The models from this study showcase that genetic summary statistics such as allelic richness and *F*st are good predictors of IUCN status and should be included in species extinction probability assessments.

## Author Contributions

**Anais Aoki:** conceptualization (equal), data curation (equal), formal analysis (equal), investigation (equal), methodology (equal), software (equal), validation (equal), visualization (equal), writing – original draft (equal), writing – review and editing (equal). **Arun Sethuraman:** conceptualization (equal), data curation (equal), formal analysis (equal), funding acquisition (equal), investigation (equal), methodology (equal), project administration (equal), software (equal), supervision (equal), validation (equal), writing – original draft (equal), writing – review and editing (equal).

## Conflicts of Interest

The authors declare no conflicts of interest.

## Supporting information


**Appendix S1:** ece372157‐sup‐0001‐AppendixS1.docx.

## Data Availability

All scripts for statistical analyses, ML‐based prediction, data visualization, as well as associated data tables, and references included in this manuscript are made available as congen.tar.gz via Zenodo (https://doi.org/10.5281/zenodo.16762445) which contains: (1) Data files collated from DataDryad for meta‐analysis that include: fish_msat, amphibians_msat, reptiles_msat, mammals_msat, and birds_msat, (2) “Violin_plots.rmd” file: code for violin plots to visualize the data distribution for each species class (Aves, Amphibia, Actionopterygii, Mammalia & Reptilia) for studies collated from the repository DataDryad used in this project, and (3) “IUCN_ML_Combined.RDM” file: code for the random forest algorithm using the entire data set (Data Dryad + Macropopgen) as one model.
