# Supplementary material for: Accurate Predictive Modeling of Conservation Status in Animal Species Using Supervised Learning
Source: Ecol Evol. 2025 Sep 15;15(9):e72157. doi: 10.1002/ece3.72157 (PMC12434340; doi:10.1002/ece3.72157)

**Supplementary Figures**

Supplemental Figure 1: Distributions of genetic diversity (observed heterozygosity, allelic richness, and mean number of alleles) and genetic differentiation (fixation index, Fst) across microsatellite studies collated from 2010-2022 in our literature survey, classified by IUCN RedList rankings (Least Concern – Critically Endangered). Distributions are broken down by class of animals (Mammalia, Aves, Actinopterygii, Amphibia, and Reptilia). Note that data were clearly insufficiently available across Amphibia and Reptilia, indicating large gaps in conservation-related studies of these taxa.


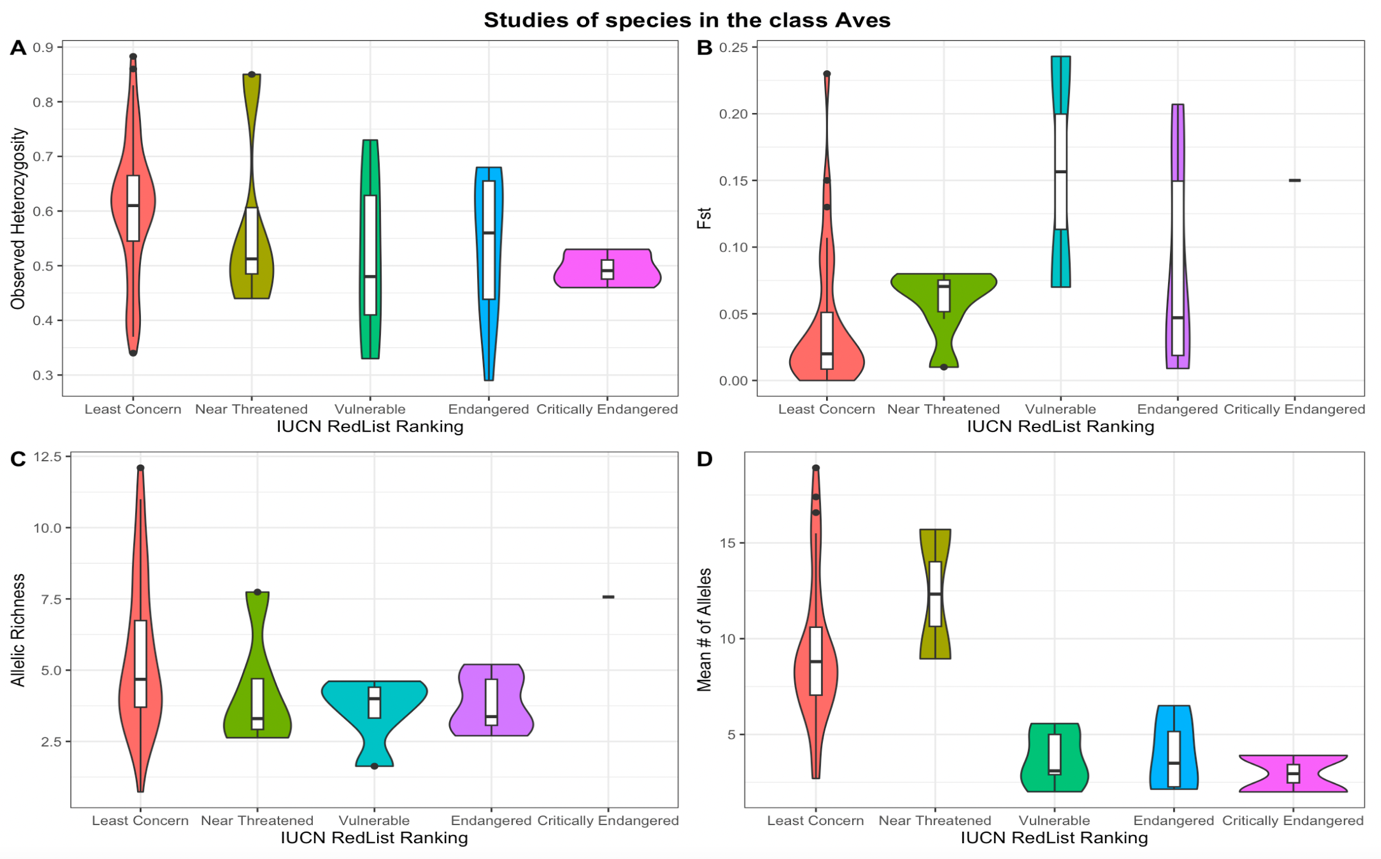


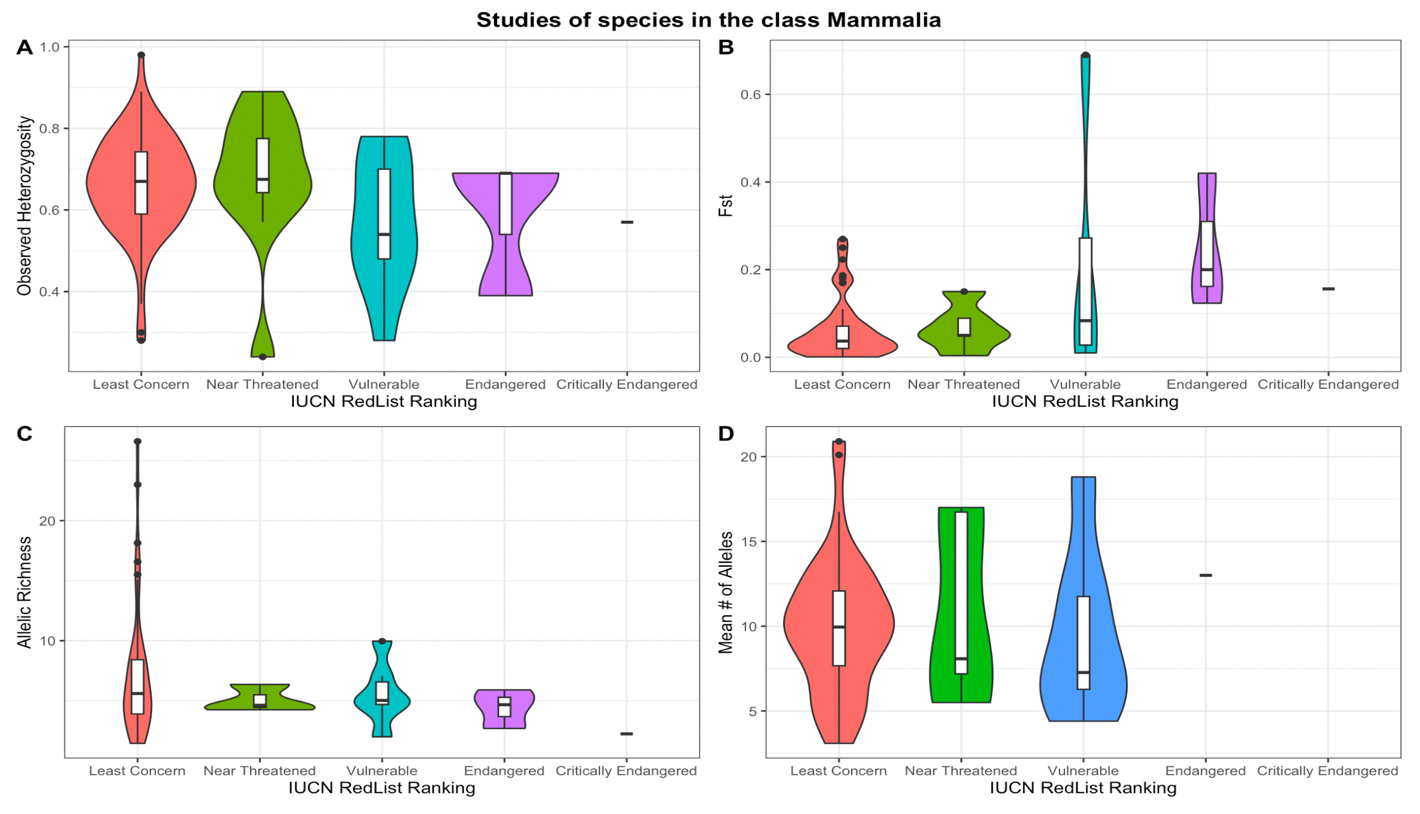


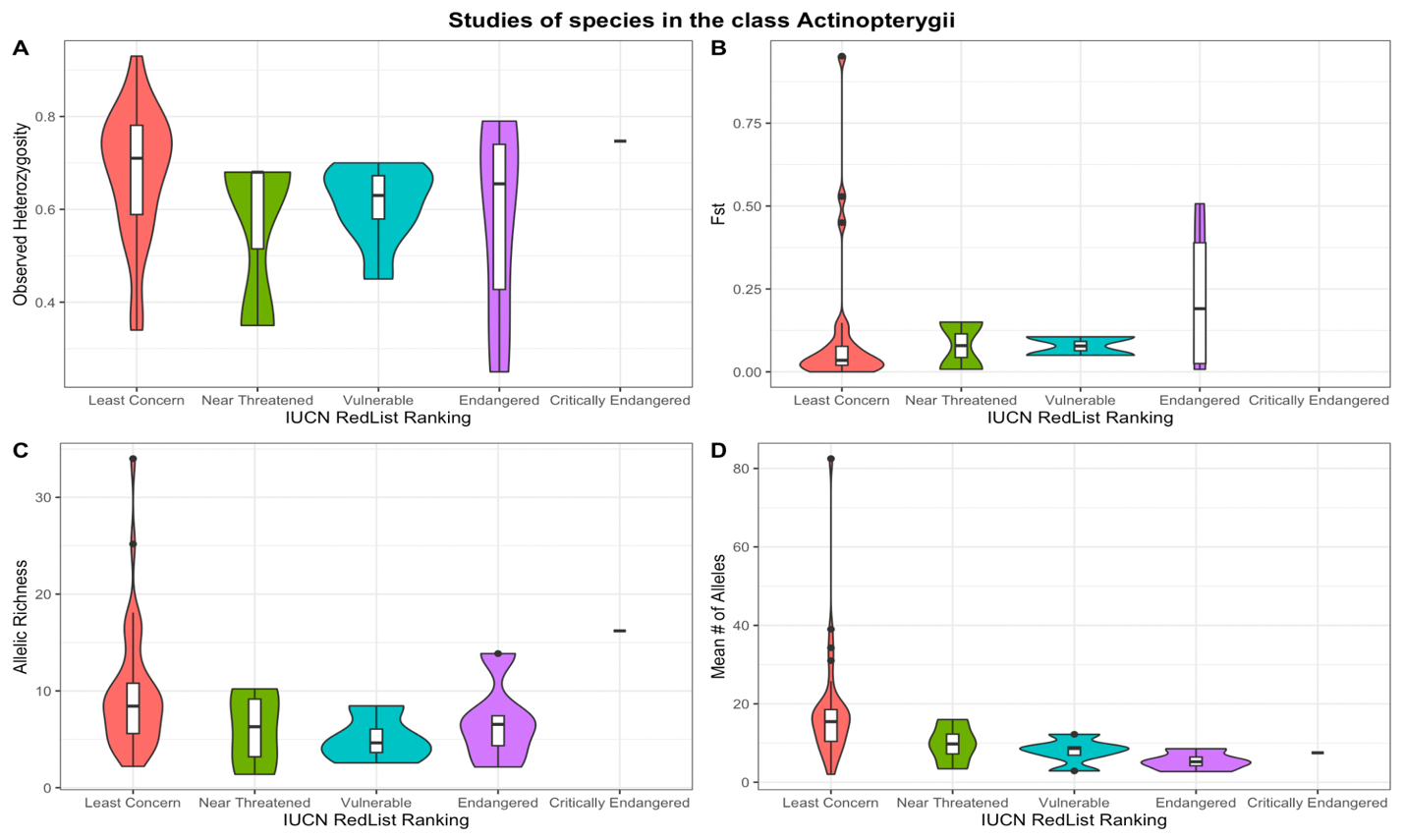


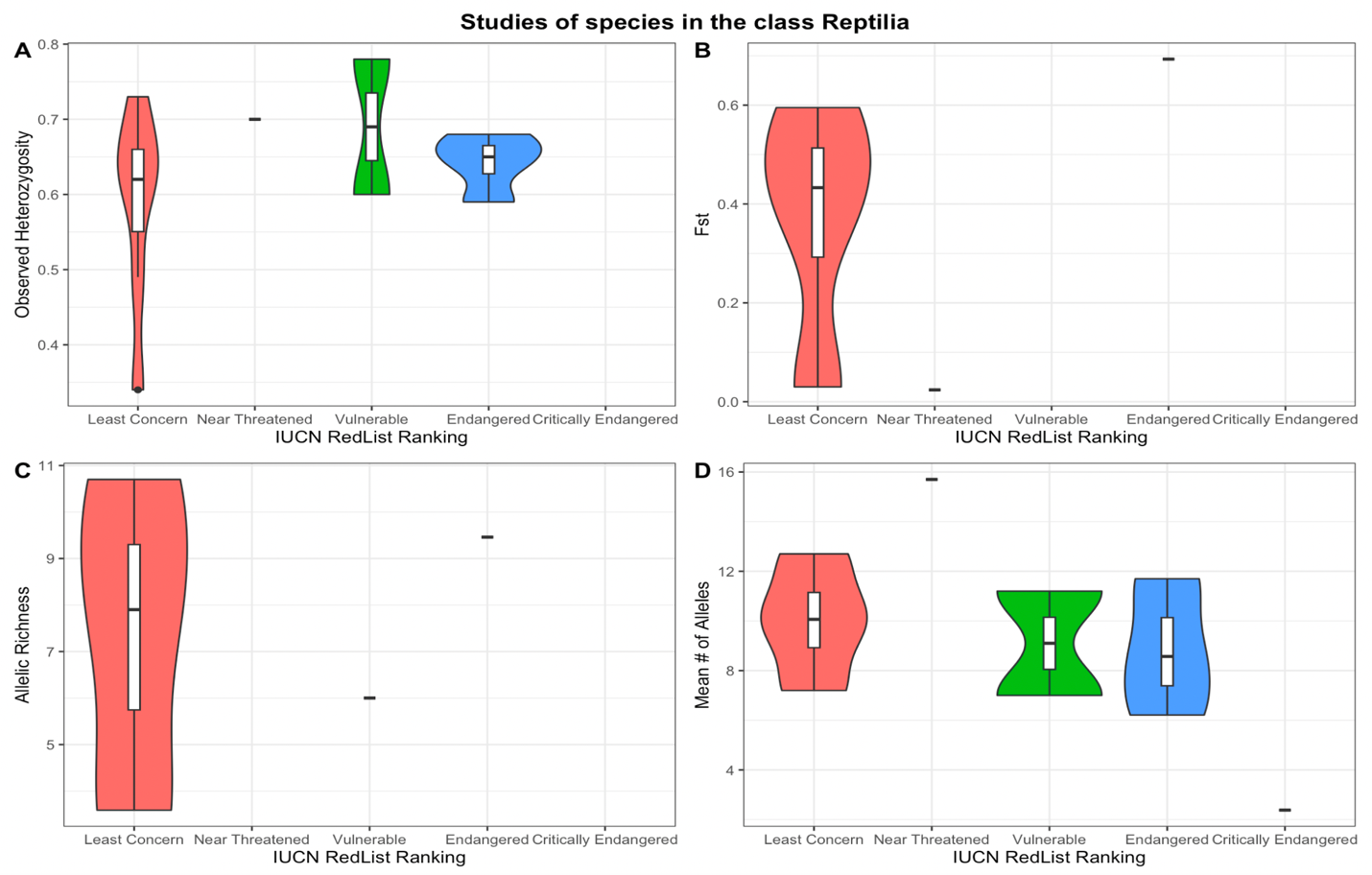


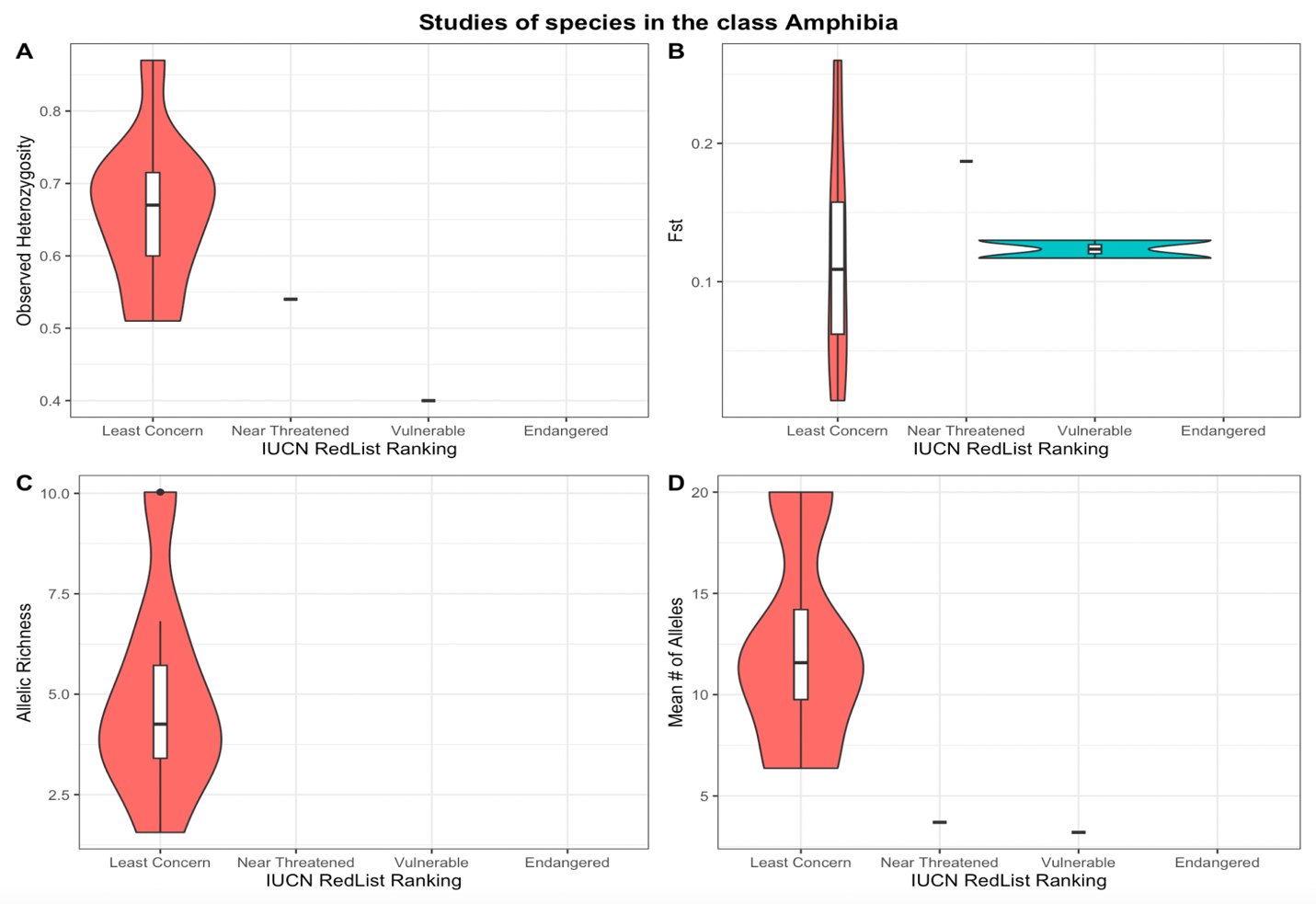


Supplemental Figure 2: Error rate of the out-of-bag (OOB) estimator and response variables (threat level) of the random forest model for class Actinopterygii.


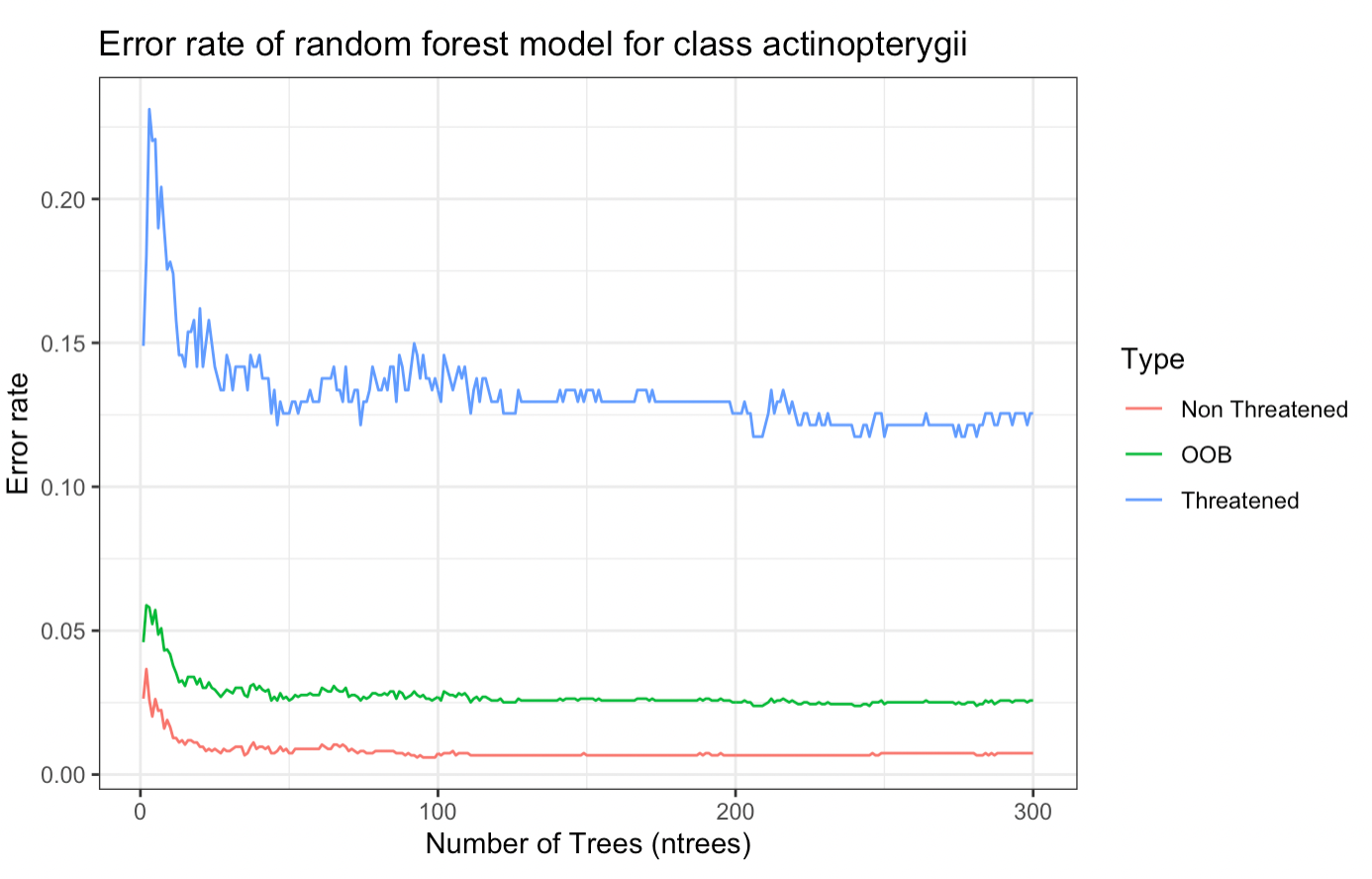


Supplemental Figure 3: Model attributes that contribute to the greatest mean decrease in accuracy in the random forest model of threat level for class Actinopterygii.


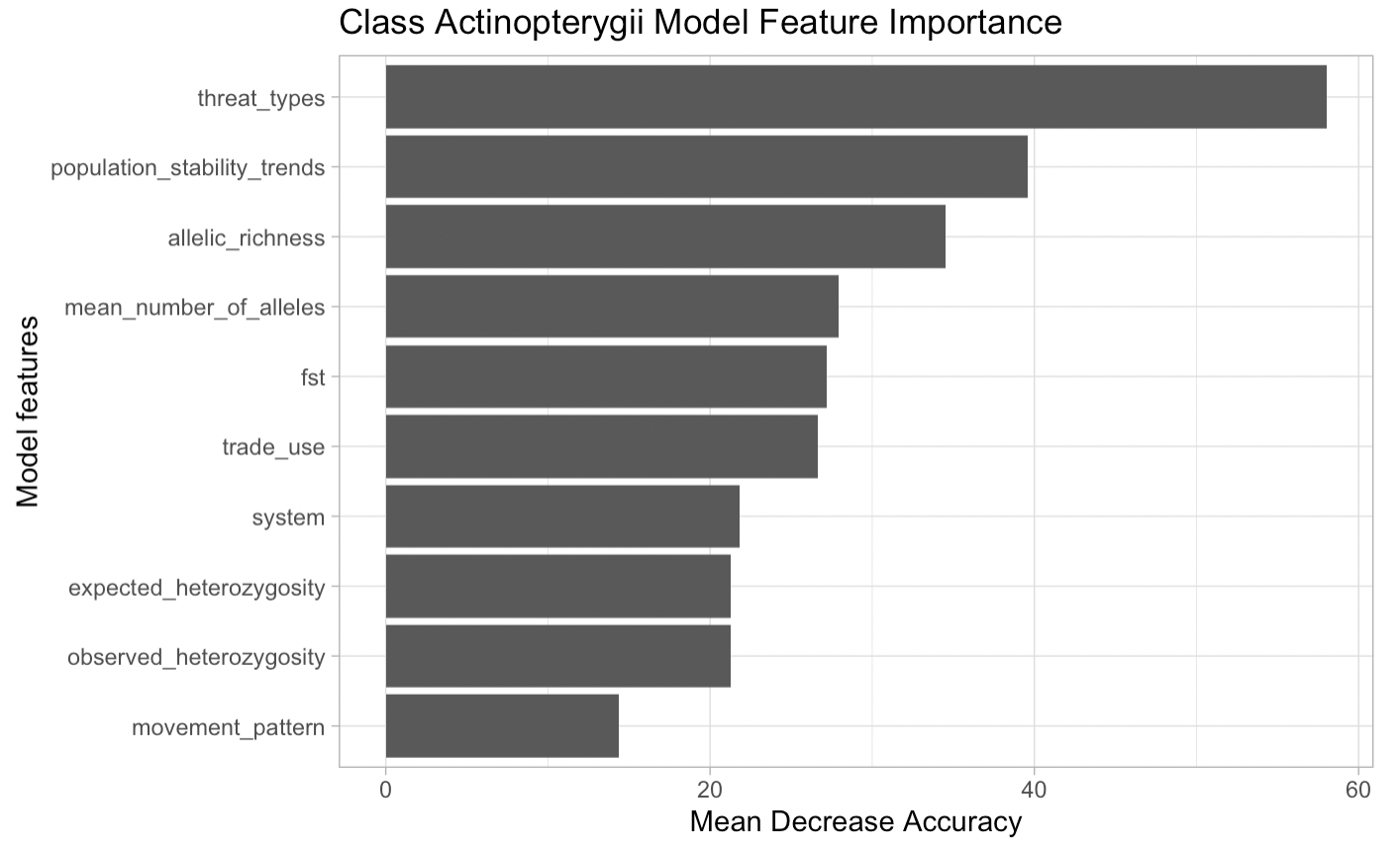


Supplemental Figure 4: Error rate of the out-of-bag estimator (OOB) and response variables (threat level) of the random forest model for class Amphibia.


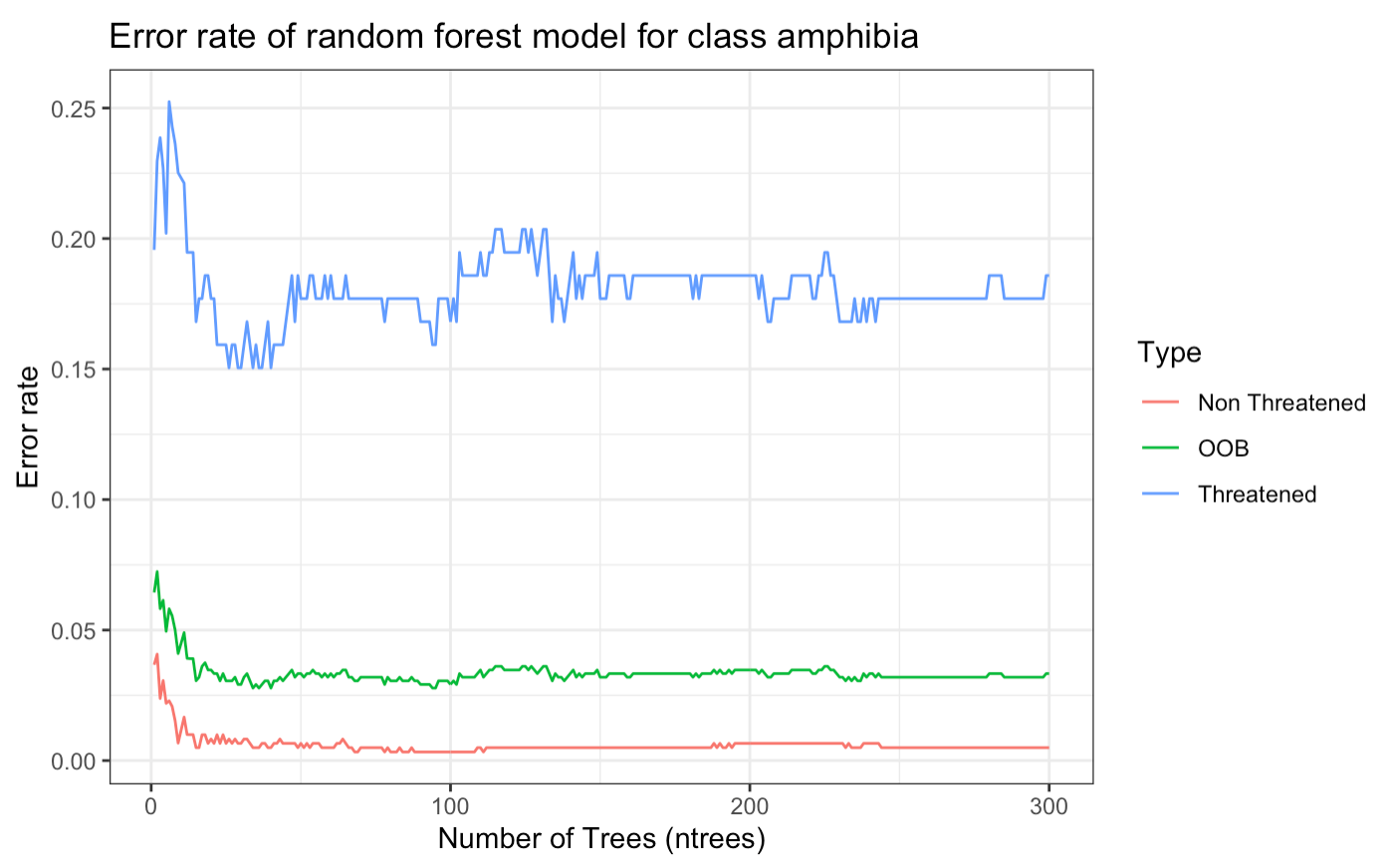


Supplemental Figure 5: Model attributes that contribute to the greatest mean decrease in accuracy in the random forest model of threat level for class Amphibia


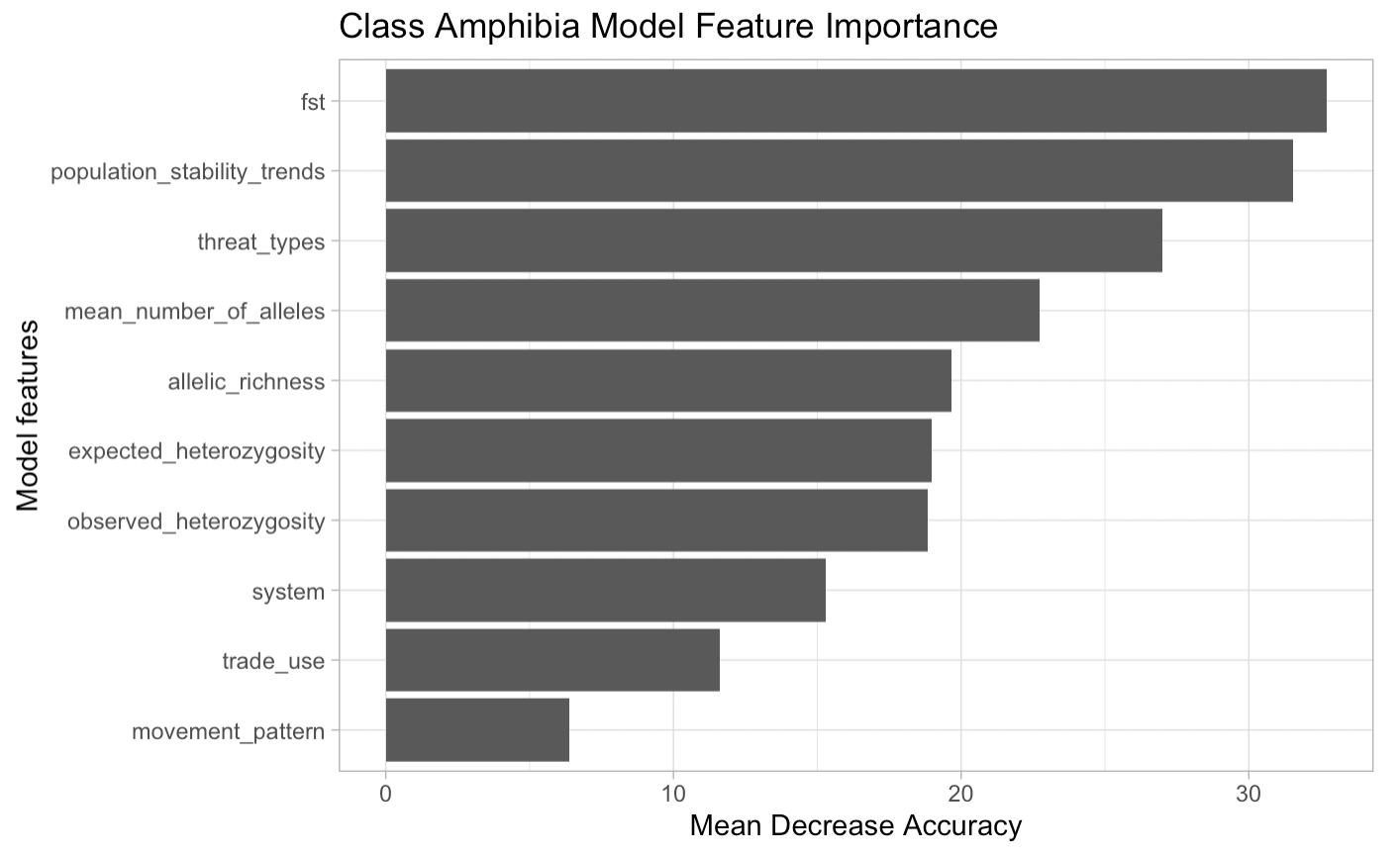


Supplemental Figure 6: Error rate of the out-of-bag (OOB) estimator and response variables (threat level) of the random forest model for class Aves.


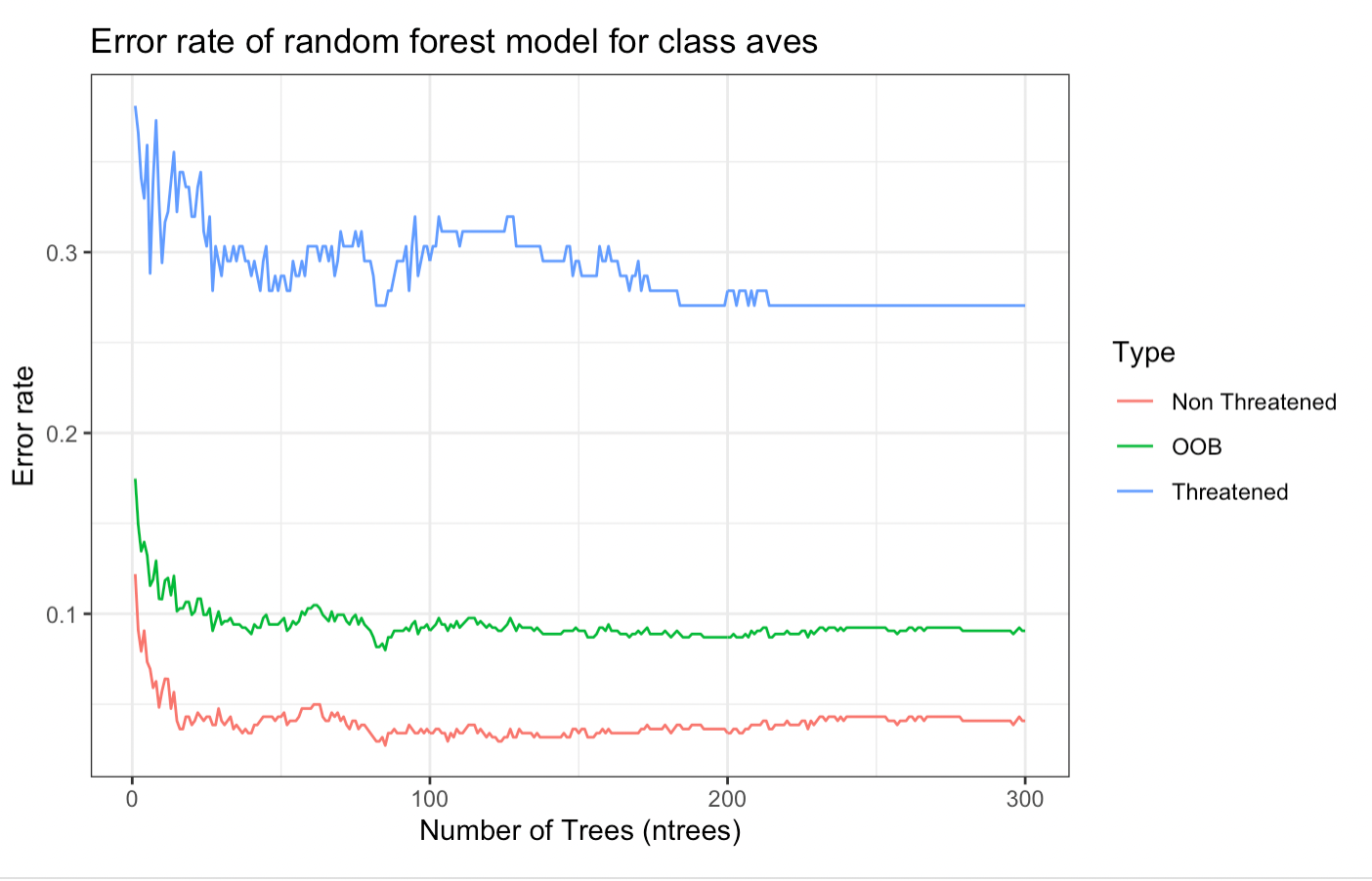


Supplemental Figure 7: Model attributes that contribute to the greatest mean decrease in accuracy in the random forest model of threat level for class Aves.


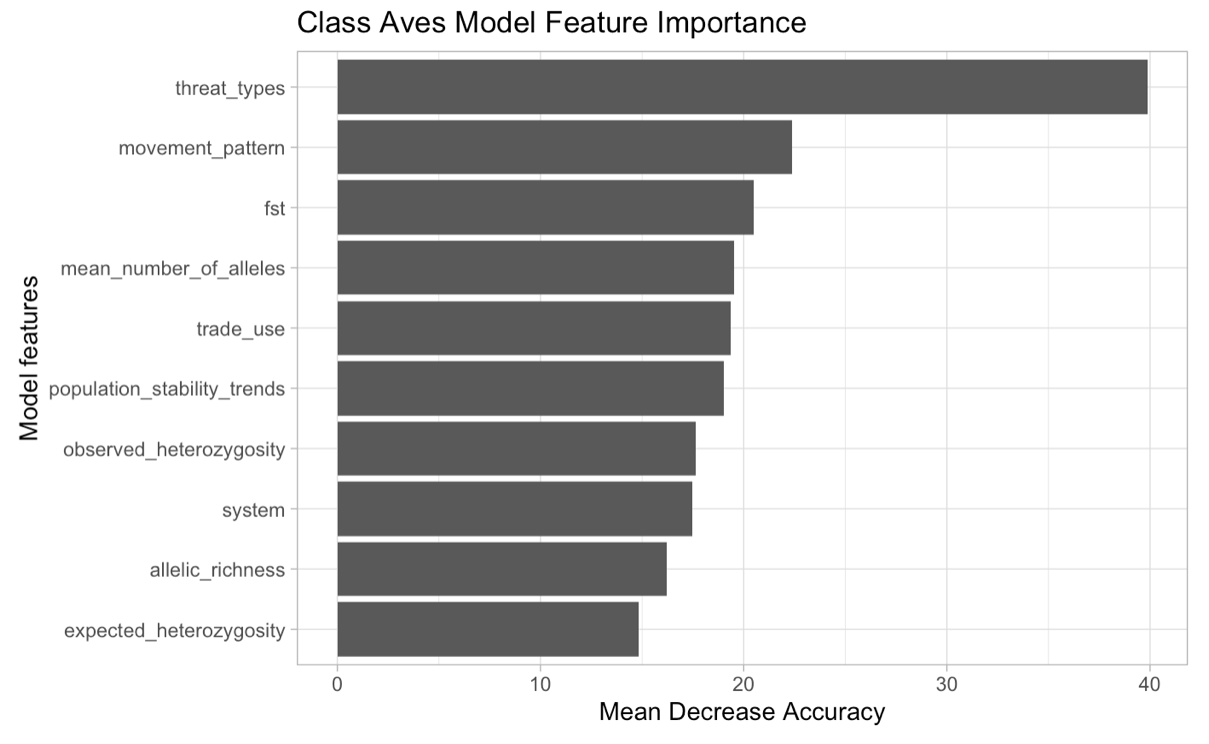


Supplemental Figure 8: Error rate of the out-of-bag estimator and response variables (threat level) of the random forest model for class Mammalia.


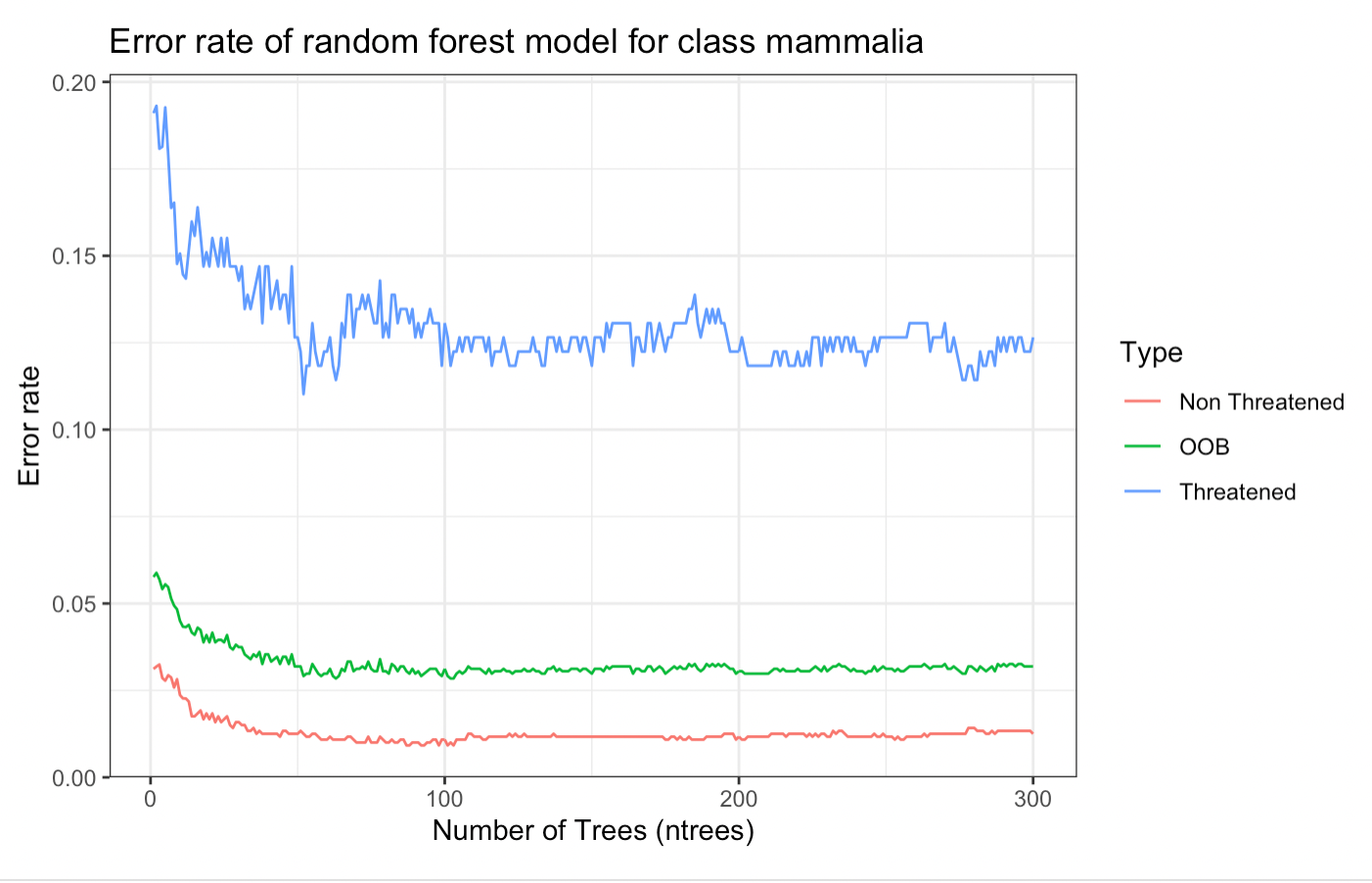


Supplemental Figure 9: Model attributes that contribute to the greatest mean decrease in accuracy in the random forest model of threat level for class Mammalia.


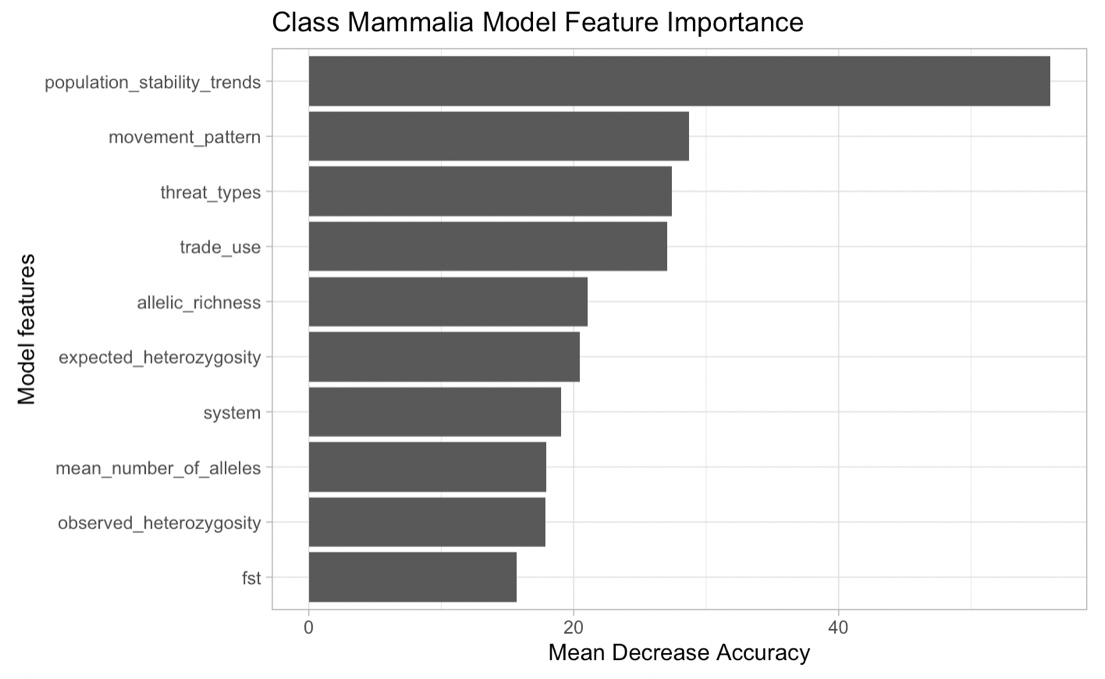


Supplemental Figure 10: Error rate of the out-of-bag (OOB) estimator and response variables (threat level) of the random forest model for class Reptilia.


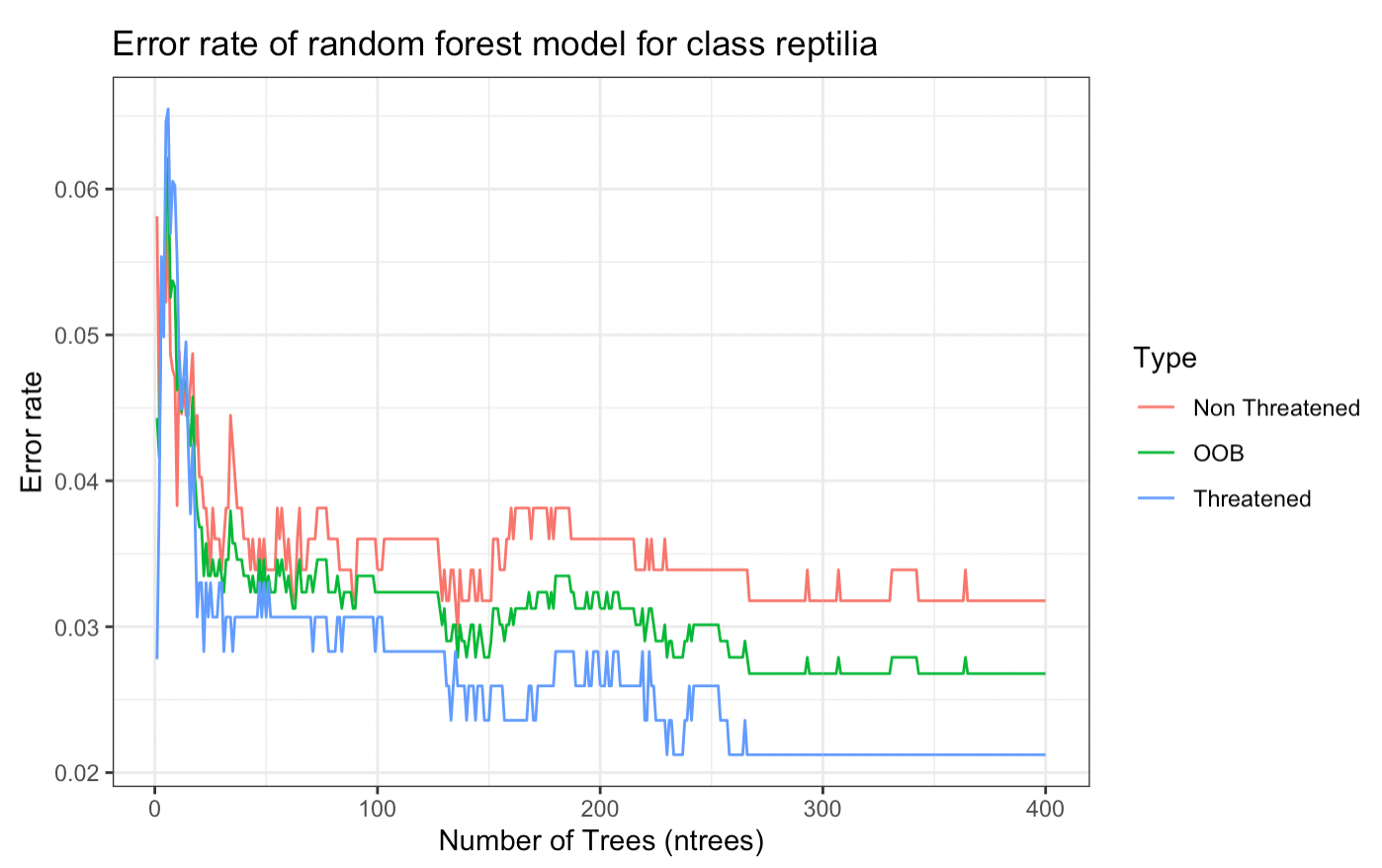


Supplemental Figure 11: Model attributes that contribute to the greatest mean decrease in accuracy in the random forest model of threat level for class Reptilia.


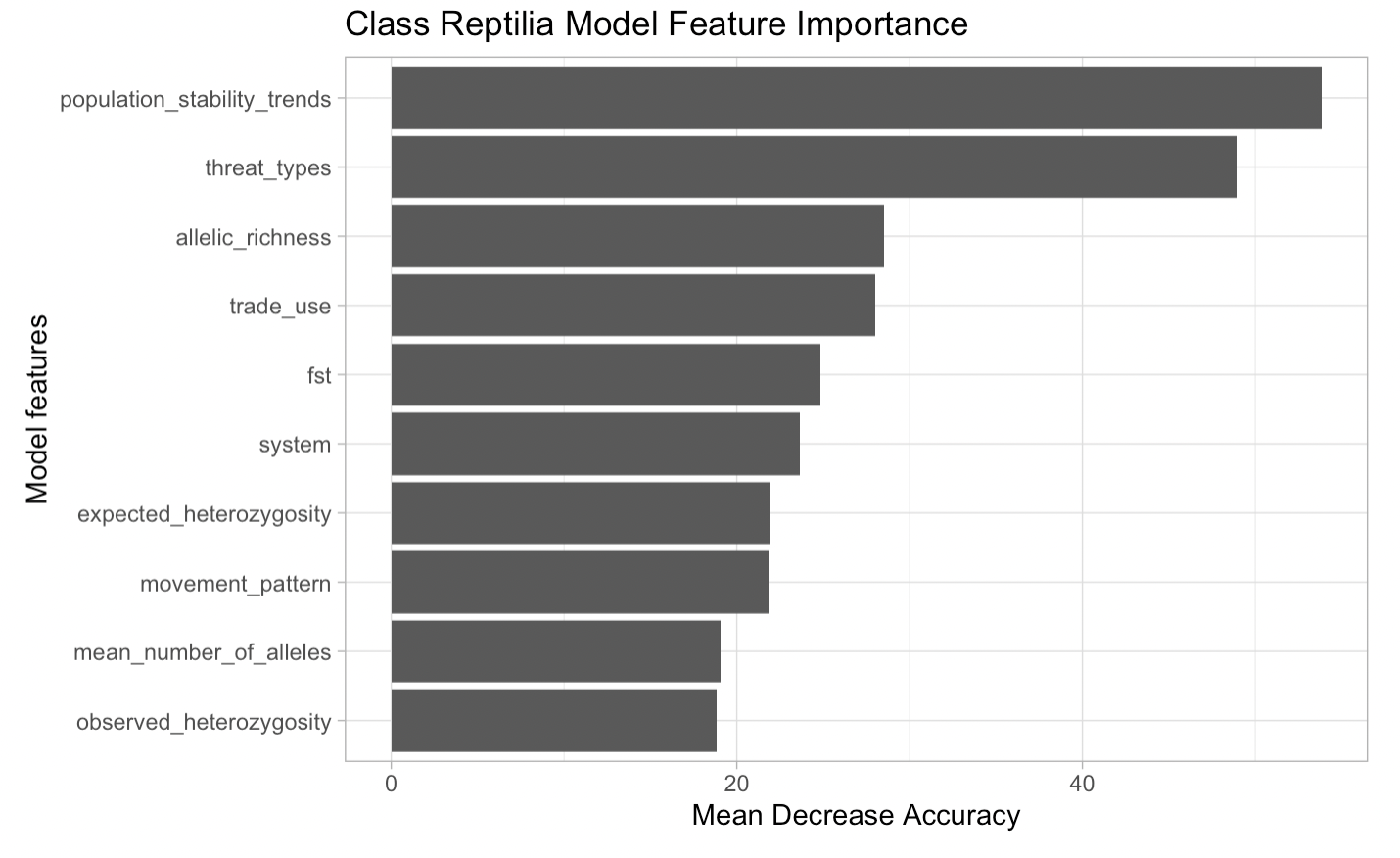

Supplement: Supplementary file 1 — Appendix S1: ece372157‐sup‐0001‐AppendixS1.docx. [file ECE3-15-e72157-s001.docx]
